# Microbial Biomarkers Differ for Various Feed Efficiency Metrics in Beef Cattle

**DOI:** 10.3390/ani15233416

**Published:** 2025-11-26

**Authors:** M. Mikayla Dycus, Utsav Lamichhane, Katherine Feldmann, Christina Welch, Andrea Osorio-Doblado, T. Dean Pringle, Todd Callaway, Jeferson Lourenco

**Affiliations:** 1 Department of Animal and Dairy Science, University of Georgia, Athens, GA 30602, USA; mmd22296@uga.edu (M.M.D.); utsav.lamichhane@uga.edu (U.L.); kfeldma2@vols.utk.edu (K.F.); cwelch@kellyreg.com (C.W.); aosoriodoblado@landolakes.com (A.O.-D.); todd.callaway@uga.edu (T.C.); 2 Department of Animal Sciences, University of Florida, Gainesville, FL 32611, USA; td.pringle@ufl.edu

**Keywords:** Angus, feed conversion, microbiome, residual average daily gain, residual feed intake

## Abstract

Cattle feed efficiency can influence operating costs and revenues, and it is a complex phenotype that can be measured by different metrices. The objective of this study was to determine which microbial taxa in the ruminal and fecal microbiomes of Angus bulls are associated with residual feed intake (RFI), residual average daily gain (RADG), feed conversion ratio (FCR), and adjusted feed conversion ratio (AFCR) by using a large sample size. The metrics RFI and RADG are calculated from the difference between an animal’s actual feed intake and actual gain, to their predicted intake and gain, respectively, and AFCR is the FCR adjusted for the animal’s size. Rumen (*n* = 1176) and fecal (*n* = 1179) samples were collected from Angus bulls from 10 contemporary groups located across the continental U.S. Animals were assigned to three classifications (High, Medium, or Low) for each feed efficiency metric within each contemporary group, according to their individual performance data. To compare the feed efficiency metrics, a cost analysis scenario was performed, and bulls classified by RADG had the greatest correlation with predicted profit (r^2^ = 0.98). For alpha diversity, only classification by FCR resulted in differences within both rumen and fecal samples. In the rumen environment, only ‘X112’ was significant (*p* = 0.02) by RFI classification, and Succinivibrionaceae was significant (*p* = 0.01) by FCR classification. In the fecal environment, a total of 19 families significantly differed, with bulls classified by RFI having the greatest number of different families. Bull feed efficiency status had greater impacts in the fecal microbiome compared to the rumen, but results should be taken in context since the animals were classified within each contemporary group. Still, differences detected within the fecal microbiome may allow for more assessable microbiome applications; however, the metric used to classify cattle as feed efficient will determine which microbial families are relevant.

## Simple Summary

This study investigated how different beef cattle feed efficiency metrics influence which microbial families are identified as potential biomarkers in the rumen and feces of Angus bulls. The results showed that a greater number of microbial families were detected in the fecal microbial environment, regardless of which feed efficiency metric was chosen. Additionally, significant fecal microbial families differed, depending on which feed efficiency metric was chosen. These findings provide insight into practical applications of identifying feed efficient animals within a herd by utilizing the animal’s gastrointestinal tract microbial composition.

## 1. Introduction

Beef production system profitability relies heavily on management practices. These practices directly influence both production costs and revenue [1]. In the United States, livestock is typically sold per hundredweight; therefore, animals that gain weight faster have the potential to generate more revenue. Feed expenses represent the greatest beef operating cost [2], underscoring feed efficiency’s importance in reducing expenses for both producers and consumers [3]. In addition to costs, there are concerns regarding environmental sustainability, which can be addressed through better feed efficiency [4]. Methane is primarily produced from methanogenic archaea within the rumen [5], and its abundance and activity may vary with feed efficiency. Enteric methane emissions can account for 2 to 12% of gross energy loss in ruminants [6], and cattle that have greater feed efficiencies have been found to have lower methane emissions [7].

Microorganisms inhabiting the ruminant gastrointestinal tract (GIT) significantly influence feed conversion efficiency due to volatile fatty acid, microbial protein, and greenhouse gas production [8,9,10]. The ruminant GIT is not one holistic microbiome, as [11] and [12] unveiled differences in foregut and hindgut microbial communities and overall diversity. Previous research using relatively small sample sizes concluded that specific microbial taxa within the GIT were linked with host traits such as feed efficiency [11,13,14]. Therefore, variation in microbial community structure may partly explain observed differences in feed efficiency among cattle. However, most studies used a limited sample size in their evaluations, making it harder to extrapolate their findings to the population level. In addition, feed efficiency is a complex phenotype that can be measured by different metrices [15], each representing different biological and mathematical efficiency components [16].

Residual feed intake (RFI) is a common metric for calculating feed efficiency in research because it allows comparison between animals differing in production level and body size [17], and it is calculated as the difference between actual and predicted feed intake based on the animal’s metabolic weight and growth over a period, where a smaller RFI is more desirable [18]. For example, cattle with a negative RFI value consume less feed than expected for their growth rate, indicating higher efficiency. Residual average daily gain (RADG) is the difference between actual and predicted weight gain based on the animal’s metabolic weight and feed intake [19]. Cattle with greater RADG are more profitable, and this metric is the current feed efficiency selection tool used by the American Angus Association [20]. Feed conversion ratio (FCR) is a simpler, traditional feed efficiency metric defined as the amount of feed consumed on a dry matter basis divided by the amount of weight gained, where a smaller FCR is more favorable [16]. Adjusted feed conversion ratio (AFCR) is FCR adjusted for the animal’s size by multiplying it to the trial group’s metabolic mid weight divided by the animal’s metabolic mid weight [21]. The Beef Improvement Federation endorses AFCR as a proper metric to evaluate feed conversion efficiency [22].

As indicated, there are multiple ways to assess feed efficiency in beef cattle, each having advantages and limitations. Moreover, there is no consensus on how to classify animals as efficient or non-efficient for feed conversion. This study aimed to assess ruminal and fecal microbiomes in a large group of Angus bulls (n = 1179), and to correlate the findings with four distinct feed efficiency metrics: RFI, RADG, FCR, and AFCR. The goal of the current study was to determine which microbial families found in the rumen and feces were associated with each feed efficiency metric calculated. Additionally, we anticipated that the use of a large sample size would enhance statistical robustness, allowing us to establish more conclusive links between beef cattle’s GIT microbiome composition and feed efficiency. We hypothesized that microbial families in the rumen and feces of yearling Angus bulls would differ among feed efficiency metrics.

## 2. Materials and Methods

### 2.1. Feed Efficiency Testing Center and Bull Selection

In collaboration with Angus Genetics Inc. (St. Joseph, MO, USA), feed efficiency testing centers were selected based on their ability to record individual feed intake of bulls, utilizing systems validated by the American Angus Association (St. Joseph, MO, USA). Based on logistics and cattle availability, 1460 yearling Angus bulls (11 to 18 months of age) were selected for the study. Samples from animals that were not registered with the American Angus Association and those that did not meet quality standards for microbiome analysis were removed, so the study proceeded with a total of 1176 rumen samples and 1179 fecal samples. Samples from 10 contemporary groups (A, B, C, D, E, F, G, H, I, and J) were collected from seven commercial feed efficiency testing centers located in Georgia, Iowa, California, Nebraska, and Montana and one research herd maintained in West Virginia. One of the testing centers in Iowa and one in Montana were sampled twice, during two separate feed efficiency testing periods. On average, each feed efficiency testing center had 118 bulls (454 ± 142 kg), and all sample collections took place from June 2022 through January 2023. Bulls used in the study were adapted to their feed ration (Table S1) for at least two weeks prior to sample collection. Bulls were subjected to feed efficiency testing for approximately 60 days. Individual feed intake and performance data were obtained, and four feed efficiency metrices were calculated from the gathered data: RFI, RADG, FCR, AFCR. The calculated value for each metric was then ranked from greatest to least within each testing center contemporary group. The upper 10% of animals were classified as High, the lower 10% as Low, and the middle 80% as Medium.

### 2.2. Feed Efficiency Calculations

Average daily gain (ADG) of each bull was calculated by the equation:

ADG=(start BW−end BW)/DOT

In which, start body weight (BW) = bull’s weight at the beginning of the feed testing period, end BW = bull’s weight at the end of the feed testing period, and DOT = days on test.

Mid-test metabolic body weight (MBW) was calculated by the equation as follows:

$$MBW={\left[(start\;BW+end\;BW)/2\;\right]}^{0.75}$$

Predicted dry matter intake (PDMI) was estimated by the linear regression coefficient as follows:

$$PDMI=\;\alpha +\left(\beta_{1}\;\times \;ADG\right)+\left(\beta_{2}\;\times \;MBW\right)\;+\;\varepsilon$$

In which α = intercept of the regression equation, $\beta_{1}$ and $\beta_{2}$ = linear regression coefficients, and ε = random error term.

Then, RFI was calculated as the differences between the calculated PDMI and the actual dry matter intake (DMI), as recorded from the individual feed intake monitoring systems.

RFI=DMI−PDMI

Predicted average daily gain (PADG) was estimated by the linear regression coefficient as follows:

$$PADG=\;\alpha +\left(\beta_{1}\;\times \;DMI\right)+\left(\beta_{2}\;\times \;MBW\right)\;+\;\varepsilon$$

In which α = intercept of the regression equation, $\beta_{1}$ and $\beta_{2}$ = linear regression coefficients, and ε = random error term.

Then, RADG was calculated as the differences between the calculated PADG and the calculated average daily gain (ADG):

RADG= ADG−PADG

To calculate FCR, the bull’s DMI was divided by ADG, as follows:

FCR= DMI/ADG

For AFCR, FCR is multiplied by the trial group’s mid-test MBW divided by the individual bull’s MBW, as calculated using the following equation:

AFCR= FCR × (group MBW/ individual MBW)

### 2.3. Collection of Ruminal and Fecal Samples

Collection of GIT samples followed procedures described by [23]. Briefly, ruminal fluid contents were collected via esophageal tubing and vacuum pump, and the first collection from each bull was discarded to eliminate saliva contamination. Once the second sample was collected, rumen fluid was homogenized by hand swirling and approximately 10 mL was placed in sterile conical tubes. Through rectal palpation, fresh fecal samples were collected and transferred to sterile 15 mL conical tubes. Immediately following collection, both ruminal and fecal samples were flash frozen by immersion in liquid nitrogen, placed in dry ice, shipped in dry ice to the University of Georgia Animal and Dairy Science Department (Athens, GA, USA), and stored at −80 °C until further analysis.

### 2.4. DNA Extraction and Sequencing

Although samples were collected over a wide period, all samples were analyzed once collections were completed to avoid extraction variation. Deoxyribonucleic acid (DNA) was extracted from samples following procedures adapted from [24], which used mechanical and enzymatic methods. Briefly, ruminal fluid (0.35 mL) and fecal samples (0.35 g) were transferred into a 2 mL lysing matrix E tube (MP Biomedicals LLC, Irvine, CA, USA). Sample mechanical disruption was accomplished using a FastPrep-24 5G homogenizer (MP Biomedicals, LLC, Irvine, CA, USA) at 6.0 m/s for 40 s, rested for 20 s, and repeated at 6.0 m/s for 40 s. A QIAamp Fast DNA Stool Mini Kit (QIAGEN, Venlo, The Netherlands) was used for enzymatic extraction and DNA purification. Final concentration was checked via fluorometry (Qubit; Thermo Fisher Scientific, Waltham, MA, USA). Samples with concentrations less than 5 ng/µL were discarded, and the DNA extraction process was repeated. Negative controls (PCR grade water) were randomly included in DNA extraction and library preparation, to ensure there was no cross-contamination. Additionally, commercial mock communities were also randomly included and served as positive controls, to validate DNA extraction and library preparation techniques (ZymoBIOMICS^TM^ Microbial Community DNA Standard and ZymoBIOMICS^TM^ Microbial Community Standard, respectively).

Amplicon libraries were generated by two rounds of polymerase chain reaction (PCR) amplifications as outlined by the Illumina 16S Metagenomic Sequencing Library Preparation guide. The first round of PCR amplification targeted the V3 and V4 hypervariable regions of the 16S rRNA gene with the forward: S-D-Bact-0341-b-S-17 (5′-CCTACGGGNGGCWGCAG-3′) and reverse: S-D-Bact-0785-a-A-21 (5′-GACTACHVGGGTATCTAA TCC-3′) primer pairs [25], followed by PCR clean-up using AMPure XP beads (Beckman Coulter Life Sciences, Indianapolis, IN, USA). A second PCR step was performed to attach Illumina’s indices and sequencing adapters (Nextera XT Index Kit; Illumina Inc., San Diego, CA, USA), followed by a second PCR clean-up step using AMPure XP beads. The final library was quantified via fluorometry (Qubit; Thermo Fisher Scientific, Waltham, MA, USA).

Samples were delivered to the Kelly Products Inc. sequencing lab (Covington, GA, USA) for 16S rRNA gene sequencing. Sequencing was performed using an Illumina MiSeq v3 2 × 300 bp kit (Illumina Inc., San Diego, CA, USA). A well-characterized bacteriophage PhiX genome (PhiX Control v3 Library; Illumina Inc., San Diego, CA, USA) was used as a control for sequencing runs.

### 2.5. Bioinformatic Analysis

Sequence data (FASTQ files) were demultiplexed and imported into QIIME2 v.2023.2 [26,27]. The DADA2 plugin was used to control sequence quality, merge forward and reverse reads, and remove chimeric sequences [28]. The feature-classifier plugin, which utilized a Naïve Bayes classifier trained on the Greengenes2 reference database [29], was used for taxonomic classification. Individual microbial taxa were then summarized as relative abundance at different taxonomic levels, and the family, genus, and species levels were further investigated with statistical analysis. All taxa that were less abundant than 0.1% were classified as ‘Other.’ In addition, microbial richness, diversity, and evenness (Pielou’s Evenness) were calculated.

### 2.6. Cost Analysis Scenario

A theoretical calculation of daily feeding costs, daily financial return, and profit was carried out to project how each animal enrolled in the study performed, and to evaluate how each feed efficiency metric influences profit margin. To estimate daily feeding cost, daily DMI was multiplied by a fixed cost of USD 0.4851/kg dry matter (DM). Indirect costs such as yardage, health, and labor were included in the calculation in the fixed cost per kg of DM. Then, ADG was multiplied by a fair market value of USD 4.4101/kg to estimate the daily financial return. The fair market value was based on averages from the United States Department of Agriculture Agricultural Marketing Service livestock auction summary. Profit was then obtained by subtracting the financial return minus the daily feeding cost.

### 2.7. Statistical Analyses

Statistical analyses were performed separately for the ruminal and fecal samples using the software Minitab^®^ v22.1.0 (Minitab LLC, State College, PA, USA). Individual microbial taxa were analyzed by fitting a mixed-effects model in which DNA sequencing batch served as a random factor and the testing center contemporary group (TC), feed efficiency classification (Class), and their interaction (TC × Class) served as the fixed factors. Due to significance, the interaction term was included in the mixed-effects model. The means of Class and TC × Class were separated using Tukey’s Honest Significant Difference test at a confidence level of 95%. Animal performance data and microbial richness, diversity, and evenness indexes were analyzed similarly. Correlations among the four feed efficiency metrices and calculated profit were determined by a linear regression model. For all statistical tests, results were considered significant at *p* ≤ 0.05, and trends were recognized at 0.05 < *p* ≤ 0.10.

## 3. Results

### 3.1. Animal Performance

Daily DMI, ADG, and feed conversion (F:G) for bulls classified as High, Medium, and Low for RFI, RADG, FCR, and AFCR, are displayed in Table 1. Bulls classified by RFI, FCR, and AFCR differed in DMI (*p* < 0.01), but bulls classified by RADG did not differ (*p* = 0.66) from each other. Bulls classified by RFI, RADG, FCR, and AFCR differed in terms of ADG (*p* ≤ 0.04). Bulls classified by RADG, FCR, and AFCR differed in terms of feed conversion (*p* < 0.01), and bulls classified by RFI tended to differ (*p* = 0.08). High-RFI bulls consumed 3.37kg more per day when compared to Low-RFI (*p* < 0.01), but High-RFI bulls did not differ (*p* = 0.48) in terms of ADG when compared to Low-RFI bulls. High-RFI bulls tended to have a greater (*p* = 0.72) feed conversion when compared to Low-RFI bulls. Bulls classified by RADG did not differ (*p* = 0.66) in terms of DMI. High-RADG bulls gained 0.81kg more per day (*p* < 0.01) when compared to Low-RADG; therefore, Low-RADG bulls had a greater (*p* < 0.01) feed conversion when compared to both High- and Medium-RADG bulls. High-FCR bulls consumed 1.6kg more per day (*p* < 0.01) when compared to Low-FCR, and High-FCR bulls gained 0.65kg less per day (*p* < 0.01) when compared to Low-FCR bulls., leading High-FCR bulls to have a greater (*p* < 0.01) feed conversion when compared to both Low- and Medium-FCR bulls. Medium-AFCR bulls had the greatest DMI when compared to High- and Low-AFCR (*p* < 0.01) and High- and Low-AFCR bulls did not differ from each other (*p* = 0.90). However, High-AFCR bulls gained 0.79kg less per day (*p* < 0.01) when compared to Low-AFCR, and High-AFCR bulls had a greater (*p* < 0.01) feed conversion when compared to both Low- and Medium-AFCR bulls.

### 3.2. Alpha Diversities

**Figure 1:**
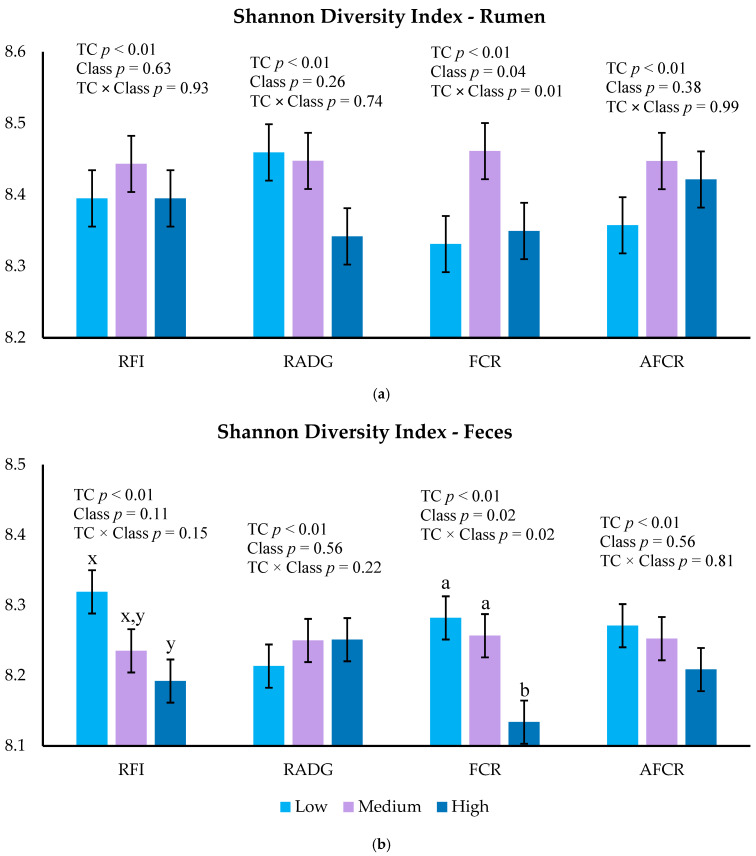
Shannon diversity index for the rumen (**a**) and feces (**b**) environment of bulls classified as High (upper 10%), Medium (middle 80%), or Low (lower 10%) feed efficiency classification (Class) for each metric. ^a,b^ Means within each feed efficiency metric with different letters differ (*p* ≤ 0.05). ^x,y^ Means within each feed efficiency metric with different letters tend to differ (*p* ≤ 0.10). TC; testing center contemporary group.

In the rumen environment, there was no RFI, RADG, or AFCR Class effect on Shannon diversity index (*p* ≥ 0.26). There was no RADG Class effect for microbial evenness or diversity (*p* > 0.26), but there was a tendency for richness (*p* = 0.08). Low-RADG bulls tended to have more richness (*p* = 0.06) than High-RADG and Medium-RADG did not differ from High- or Low-RADG (*p* > 0.24). There was no FCR Class effect for microbial evenness (*p* = 0.11), but there was an effect for richness (*p* = 0.02) and diversity (*p* = 0.04). Low-FCR bulls had less microbial richness (*p* = 0.02) when compared to Medium-FCR but did not differ (*p* = 0.38) from High-FCR. Medium- and High-FCR richness did not differ from each other (*p* = 0.60). Low-FCR bulls had the least numerical microbial diversity, but the means did not differ (*p* > 0.11) from each other when analyzed by Tukey’s Honest Significant Difference test. Ruminal microbial diversity for each feed efficiency metric is shown in Figure 1a, and rumen alpha diversity metrics are shown in Table S2.

In the fecal environment, there was no AFCR and RADG Class effect for alpha diversity indices (*p* > 0.05). There was no RFI Class effect for microbial richness (*p* = 0.12), evenness (*p* = 0.37), or diversity (*p* = 0.11), but Low-RFI tended to be more diverse and richer (*p* ≤ 0.10) than High-RFI and neither differed from Medium-RFI (*p* > 0.27). There was no FCR Class effect for microbial richness (*p* = 0.14), but there was an effect for evenness (*p* = 0.04) and diversity (*p* = 0.02). High-FCR bulls had less microbial evenness (*p* = 0.04) when compared to Medium-FCR but only tended to be less (*p* = 0.07) than Low-FCR. Medium- and Low-FCR bulls did not differ in terms of evenness (*p* = 0.86). High-FCR bulls had less microbial diversity compared to Medium- (*p* = 0.02) and Low-FCR bulls (*p* = 0.04), and Medium- and Low-FCR bulls tended to differ (*p* = 0.85) from each other. Fecal microbial diversity for each feed efficiency metric is shown in Figure 1b, and fecal alpha diversity metrics are shown in Supplementary Table S3.

### 3.3. Microbial Abundance

In the rumen environment, there were two families (‘X112’ and Succinivibrionaceae) with significantly different abundance due to feed efficiency classification in the four feed efficiency metrices (Table 2). Bulls classified by RFI significantly differed in the taxa ‘X112’ (*p* = 0.02). ‘X112’ was less abundant in Low- and Medium-RFI bulls compared to High-RFI bulls (*p* < 0.03) but no differences were detected between Low- and Medium-RFI bulls (*p* = 0.59). Bulls classified by FCR differed in the abundance of family Succinivibrionaceae (*p* = 0.01). Moreover, there was a tendency for Paludibacteraceae (*p* = 0.09), ‘CAG-74’ (*p* = 0.08), and Fibrobacteraceae (*p* = 0.07) to be different. Succinivibrionaceae was significantly more abundant (*p* = 0.01) in the High-FCR bulls when compared to Medium-FCR but did not differ (*p* = 0.33) from Low-FCR bulls. Medium-FCR bulls did not differ (*p* = 0.48) from Low-FCR in Succinivibrionaceae. Paludibacteraceae tended to be more abundant (*p* = 0.08) in Low-FCR bulls when compared to High-FCR, but no other differences were detected. ‘CAG-74’ tended to be more abundant (*p* = 0.08) in High-FCR bulls when compared to Low-FCR, but no other differences were detected. Fibrobacteraceae tended to be more abundant (*p* = 0.06) in Medium-FCR bulls when compared to High-FCR, but no other differences were detected.

There was no AFCR Class effect for microbial taxa (*p* > 0.05) in the rumen environment. Bulls classified by RADG tended to be different in abundance of families ‘CAG-74’ (*p* = 0.06), Ruminococcaceae (*p* = 0.08), and ‘UBA1242’ (*p* = 0.09). ‘CAG-74’ was less abundant (*p* = 0.05) in High-RADG bulls when compared to Medium-RADG but did not differ (*p* = 0.25) from Low-RADG. ‘CAG-74’ did not differ (*p* = 0.96) between Medium- and Low-RADG bulls. Ruminococcaceae tended to be less abundant (*p* = 0.06) in Low-RADG bulls when compared to Medium-RADG, but no other differences were detected (*p* > 0.42). ‘UBA1242’ tended to be less abundant (*p* = 0.08) in Medium-RADG bulls when compared Low-RADG, but no other differences were detected (*p* > 0.16).

In the fecal environment, there were 19 families affected by Class when considering all four feed efficiency metrices (Table 2), and the 10 most abundant families found in the feces of the bulls are shown in Supplemental Figure S2. Bulls classified by RFI had a greater number of families differing due to Class when compared to the other efficiency metrices. Out of those, Lachnospiraceae was the only significant family shared with another feed efficiency metric (FCR). Lachnospiraceae (*p* < 0.01), Acutalibacteraceae (*p* = 0.01), Treponemataceae (*p* = 0.01), an unidentified family from the Class Clostridia_258483 (*p* = 0.04), ‘CAG-382’ (*p* = 0.04), Coprobacillaceae (*p* = 0.02), ‘UBA1242’ (*p* = 0.03), Anaeroplasmataceae (*p* < 0.01), ‘UBA644’ (*p* = 0.01), an unidentified family from the Order RFN20 (*p* = 0.02), and ‘CAG-826’ (*p* = 0.01) were different between the RFI Class, and Bacteroidaceae (*p* = 0.08), UBA932 (*p* = 0.07), Acidaminococcaceae (*p* = 0.10), and Desulfovibrionaceae (*p* = 0.08) tended to differ. Lachnospiraceae, Anaeroplasmataceae, and an unidentified family from the Order RFN20 were less abundant in Low- and Medium-RFI bulls compared to High-RFI (*p* < 0.03) but did not differ (*p* > 0.68) from each other. Acutalibacteraceae was less abundant in Medium- and High-RFI bulls when compared to Low-RFI (*p* < 0.03) but did not differ (*p* = 0.21) from each other. Treponemataceae was more abundant (*p* = 0.01) in Medium-RFI bulls when compared to High-RFI and neither differed from Low (*p* > 0.05). An unidentified family from the Class Clostridia_258483 and ‘CAG-382’ were more abundant (*p* < 0.04) in Low-RFI bulls when compared to High-RFI and neither differed from Medium-RFI (*p* > 0.11). Coprobacillaceae was more abundant (*p* = 0.02) in High-RFI bulls when compared to Medium-RFI and neither differed from Low-RFI (*p* > 0.14). ‘UBA1242’ and ‘UBA644’ were more abundant in Low- and Medium-RFI bulls compared to High-RFI (*p* < 0.05) but did not differ (*p* > 0.70) from each other. ‘CAG-826’ was more abundant (*p* = 0.01) in Low-RFI bulls when compared to Medium-RFI and neither differed from High-RFI (*p* > 0.11). Bacteroidaceae tended to be less abundant in Low- and Medium-RFI bulls compared to High-RFI (*p* > 0.06) but did not differ (*p* = 0.87) from each other. ‘UBA932’ tended to be more abundant (*p* = 0.06) in Low-RFI bulls when compared to High-RFI and neither differed from Medium-RFI (*p* > 0.22). Acidaminococcaceae and Desulfovibrionaceae tended to be more abundant (*p* = 0.08) in High-RFI bulls when compared to Medium-RFI and neither differed from Low-RFI (*p* > 0.13).

Bulls classified by RADG had a Class effect in the fecal environment for ‘Oscillospiraceae,’ ‘UBA932,’ ‘Peptostreptococcaceae,’ Borkfalkiaceae, ‘Enterobacteriaceae_A,’ and Eggerthellaceae (*p* < 0.04). ‘Oscillospiraceae’ and Borkfalkiaceae were more abundant (*p* = 0.03) in High-RADG bulls when compared to Low-RADG, but neither differed from Medium-RADG (*p* > 0.06). ‘UBA932’ was less abundant (*p* < 0.01) in Low-RADG bulls when compared to High- and Medium-RADG but High- and Medium-RADG did not differ from each other (*p* = 0.36). ‘Peptostreptococcaceae’ was more abundant (*p* < 0.02) in Low-RADG bulls when compared to High- and Medium-RADG and High- and Medium-RADG did not differ from each other (*p* = 0.93). ‘Enterobacteriaceae_A’ was more abundant (*p* < 0.01) in Low-RADG bulls when compared to Medium- and High-RADG but Medium- and High-RADG did not differ from each other (*p* > 0.94). Eggerthellaceae was more abundant (*p* = 0.03) in Low-RADG bulls when compared to High-RADG but neither differed from Medium-RADG (*p* > 0.14).

Bulls classified by FCR had a Class effect for ‘Oscillospiraceae,’ ‘UBA932,’ Lachnospiraceae, ‘Peptostreptococcaceae,’ Borkfalkiaceae, Methanobacteriaceae, ‘Enterobacteriaceae_A,’ and Eggetherllaceae (*p* < 0.05) and Rikenellaceae, ‘Clostridia_222000,’ Turicibacteraceae, and Streptococcaceae tended to differ (*p* > 0.05). ‘Oscillospiraceae’ and Borkfalkiaceae were more abundant (*p* < 0.03) in Low-FCR bulls when compared to High- and Low-FCR tended to be more abundant than Medium-FCR (*p* = 0.08), but Medium- and High-FCR bulls did not differ (*p* > 0.10). ‘UBA932’ was less abundant in High-FCR bulls when compared to Low- and Medium-FCR (*p* < 0.01), and Low- and Medium-FCR bulls did not differ from each other (*p* = 0.15). Lachnospiraceae tended to be most abundant in High-FCR bulls when compared to Medium- and Low-FCR (*p* < 0.08) but Medium- and Low-FCR did not differ (*p* = 0.59) from each other. ‘Peptostreptococcaceae’ was more abundant in High-FCR bulls when compared to Medium- and Low-FCR (*p* < 0.01) but Medium- and Low-FCR did not differ (*p* = 0.97) from each other. Methanobacteriaceae was more abundant (*p* < 0.01) in High-FCR bulls when compared to Low-FCR and tended to be more abundant (*p* = 0.07) than Medium-FCR. Medium- and Low-FCR bulls did not differ (*p* = 0.15) in Methanobacteriaceae. ‘Enterobacteriaceae_A’ was more abundant (*p* = 0.01) in High-FCR bulls when compared to Medium-FCR and tended to be more abundant (*p* = 0.04) than Low-FCR. Medium- and Low-FCR bulls did not differ (*p* = 0.93) in ‘Enterobacteriaceae_A.’ Eggerthellaceae was more abundant (*p* = 0.04) in High-FCR bulls when compared to Low-FCR, and Medium-FCR did not differ from Low- and High-FCR (*p* > 0.16). Rikenellaceae was more abundant (*p* = 0.05) in Medium-FCR bulls when compared to High-FCR and neither differed from Low-FCR (*p* > 0.13). ‘Clostridiaceae_222000’ was more abundant (*p* = 0.05) in High-FCR bulls when compared to Medium-FCR and neither differed from Low-FCR (*p* > 0.15). Turicibacteraceae tended to be more abundant (*p* = 0.06) in High-FCR bulls when compared to Medium-FCR and neither differed from Low-FCR (*p* > 0.26). Streptococcaceae tended to be more abundant in High-FCR bulls when compared to Medium- and Low-FCR (*p* = 0.08) and Medium-FCR did not differ from Low-FCR (*p* = 0.77).

Bulls classified by AFCR in the fecal environment had the least number of significant families when compared to the other efficiency classifications. ‘Oscillospiraceae,’ ‘UBA932,’ ‘Peptostreptococcaceae,’ and Turicibacteraceae had a Class effect, and Acutalibacteraceae, ‘Enterobacteriaceae_A,’ and Eggerthellaceae tended to have a Class effect. ‘Oscillospiraceae’ was more abundant (*p* = 0.03) in Low-AFCR bulls when compared to High-AFCR and did not differ (*p* = 0.43) from Medium-AFCR. Medium-AFCR bulls tended to be more abundant (*p* = 0.09) than High-AFCR in ‘Oscillospiraceae.’ ‘UBA932’ was more abundant in Low- and Medium-AFCR bulls when compared to High-AFCR (*p* < 0.01) and Low- and Medium-AFCR did not differ from each other (*p* = 0.41). ‘Peptostreptococcaceae’ was less abundant in Low- and Medium-AFCR bulls when compared to High-AFCR (*p* < 0.01) and Low- and Medium-AFCR did not differ from each other (*p* = 0.97). Turicibacteraceae was more abundant (*p* < 0.01) in High-AFCR bulls when compared to Medium-AFCR and tended to differ (*p* = 0.08) from Low-AFCR. Medium- and Low-AFCR bulls did not differ (*p* = 0.88) in Turicibacteraceae. Acutalibacteraceae tended to be more abundant (*p* = 0.08) in Low-AFCR bulls when compared to High-AFCR and neither differed from Medium-AFCR (*p* > 0.21). Eggerthellaceae tended to be more abundant (*p* = 0.06) in High-AFCR bulls when compared to Low-AFCR and neither differed from Medium-AFCR (*p* > 0.15). ‘Enterobacteriaceae_A’ tended to be more abundant (*p* = 0.06) in High-AFCR bulls when compared to Medium-AFCR and neither differed from Low-AFCR (*p* > 0.52).

### 3.4. Relationship of Feed Efficiency Metrices and Profit

**Figure 2:**
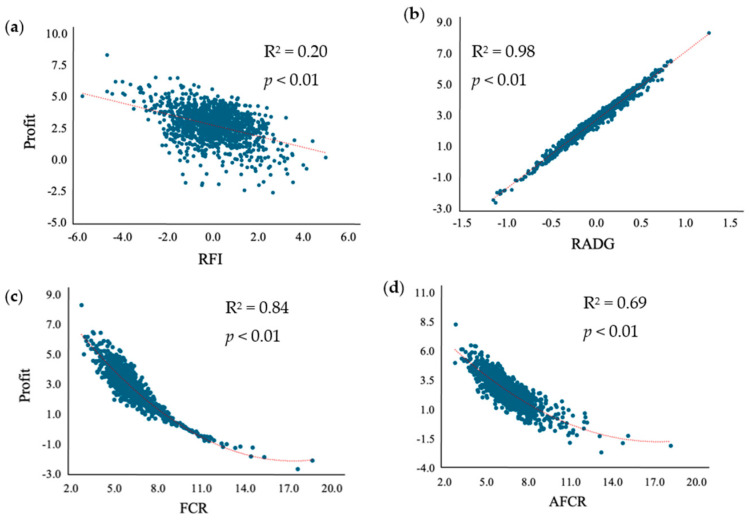
Regression of residual feed intake (RFI; (**a**)), residual average daily gain (RADG; (**b**)), feed conversion ratio (FCR; (**c**)), and adjusted feed conversion ratio (AFCR; (**d**)) efficiency metrics versus profit.

The regression of each feed efficiency metric versus profit is shown in Figure 2, and daily feed cost and financial return are shown in Figure S1. The RFI feed efficiency metric had the lowest coefficient of determination for the response variable profit, comprising only 20.2%. Conversely, the RADG metric had the best fit, with a coefficient of determination of 97.9% for profit. Feed efficiency determined by FCR and AFCR had a quadratic relationship with profit and had coefficient of determinations of 83.8% and 69.7%, respectively.

## 4. Discussion

**Figure 3:**
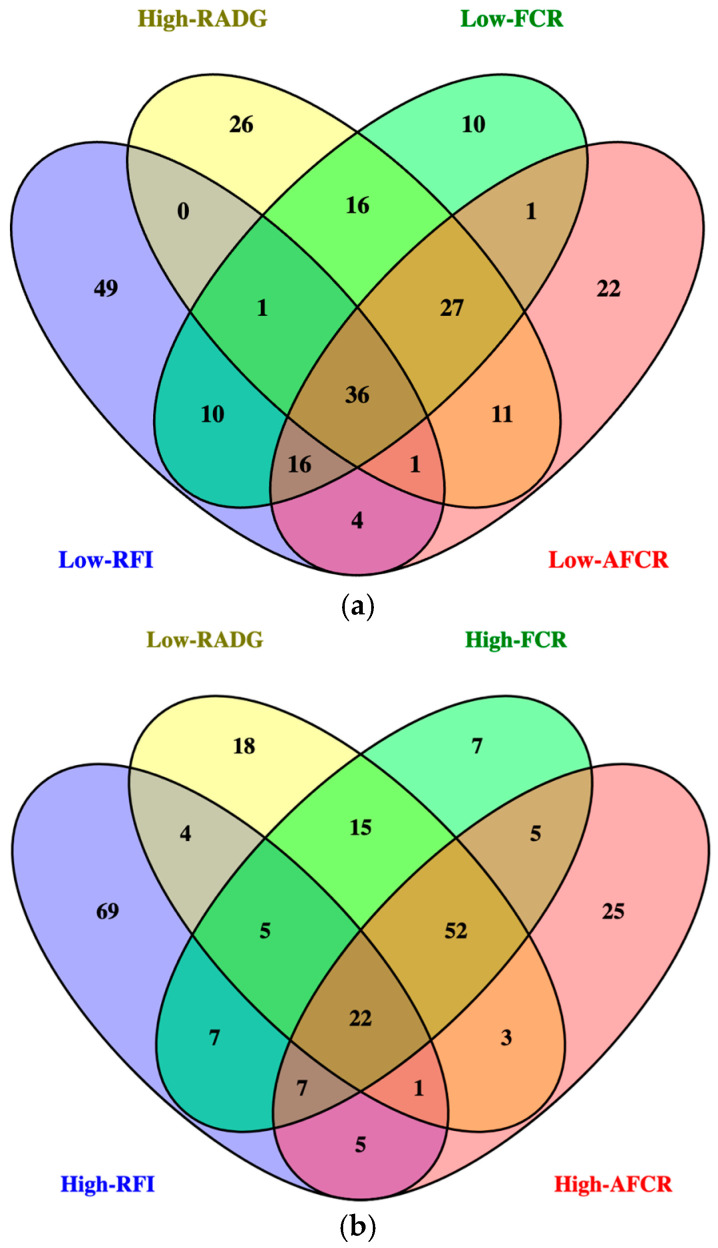
(**a**) Venn Diagram of animals that share the most desirable classification for each feed efficiency metric: residual feed intake (RFI), residual average daily gain (RADG), feed conversion ratio (FCR), and adjusted feed conversion ratio (AFCR). (**b**) Venn Diagram of animals that share the least desirable classification for each feed efficiency metric: residual feed intake (RFI), residual average daily gain (RADG), feed conversion ratio (FCR), and adjusted feed conversion ratio (AFCR).

The identification of gastrointestinal microbes that influence feed efficiency in cattle has been a topic of considerable investigation due to the magnitude of feed input costs. This has increased the use of technologies such as individual feed intake nodes to determine an individual animal’s level of feed efficiency; however, it is very expensive to obtain this data [30]. Most cattle microbiome research define efficiency by RFI, making it more assessable to compare study results and review the literature, but in doing so, other metrics are not well studied. Additionally, other efficiency metrics besides RFI are utilized in beef cattle production, so research focusing on RFI limits the practical implementation of microbiome-based feed efficiency research. In the current study, we calculated four different feed efficiency metrices: RFI, RADG, FCR, and AFCR from a large dataset of bulls fed at feed efficiency testing centers across the United States. Each feed efficiency metric is calculated differently, with emphasis put on either intake or growth; therefore, different animals are being identified as efficient for each metric. When comparing the most efficient animals for each metric, only 36 animals overlapped for all four metrics (Figure 3a). Additionally, only 22 animals shared the least efficient classification for all four metrics (Figure 3b). There were no similarities between the animals classified as being the most or least efficient for all four metrics. As a result, our findings show that the microbial families found to be significantly associated with each metric are different.

### 4.1. Animal Performance

Bulls classified as Low-RFI had the least mean DMI (9.20 kg), suggesting that reduced intake may enhance nutrient extraction efficiency. Animals with lower feed intake could potentially have lower gut passage rate, allowing for more rumination of feed, longer fermentation, and better feed degradation [31]. Additionally, [7] found that steers classified as low-RFI had up to 28% less methane production when compared to high- and medium-RFI animals, likely due to their reduction in feed intake. Furthermore, [32] reported no enteric methane differences in steers classified for RADG, potentially due to all animals having a common feed intake, regardless of RADG classification. Therefore, selecting animals with low-RFI may be the best feed efficiency metric for sustainability since it can result in lower input feed costs and lower methane emissions without negatively affecting growth performance. However, it is difficult to determine whether the differences in rumen microbiota between RFI classifications are linked to variations in feed efficiency or a consequence of feed intake functions [33].

In the current study, bulls classified as High-RADG, had the greatest mean ADG (2.04 kg/d), and there were no significant differences in DMI between Low and High-RADG bulls. Similarly, Low-FCR and Low-AFCR bulls had significantly greater ADG when compared to High bulls but did not differ in DMI. Bulls classified as Low-AFCR had the second-greatest mean ADG (2.01 kg/d). Those three feed efficiency metrices (RADG, FCR, and AFCR) select for animals that have greater outputs. Selection for RADG will yield heavier calves that will have higher nutrient requirements, making RADG an effective selection tool for feedlot cattle [34]. Further, FCR can also lead to an increase in cow size and feed intake due to its genetic correlation with growth rate [16]. When livestock is sold, the seller is paid per hundredweight because heavier animals will yield more product. The theoretical cost analysis scenario visualizes this relationship with having an almost perfect correlation (r^2^ = 0.98) between RADG and profit (financial return minus daily feed cost). Therefore, selecting animals for RADG (and to a lesser degree FCR and AFCR) may be the best feed efficiency metric for improving direct profit. Concerning genetic selection, both RFI and RADG are moderately heritable traits (0.16 to 0.43 and 0.31 to 0.41, respectively) [34,35], suggesting that producers can successfully perform selection for those traits.

### 4.2. Alpha Diversities

Lack of differences detected in the diversity of the rumen environment indicates that there are no major microbial population structure variances between animals differing in feed efficiency classifications. The results from the current study align with previous results in steers differing in ADG [36] and RFI [37]. However, numerically, the more efficient animals for every feed efficiency metric in the rumen environment had the lowest diversity. In fact, [38] found that a reduced ruminal diversity may promote the production of metabolites that are more beneficial for the productivity of the host animal.

For the fecal environment, Low- and High-RFI tended to be different in diversity (*p* = 0.10), mirroring the findings from [11], where no differences were detected in the foregut diversity, but differences were detected in the hindgut of animals classified by RFI. Nonetheless, in the current study, most of the alpha diversity metrics did not differ between efficiency classification regardless of the feed efficiency metric. Other authors also did not find differences in alpha diversity between heifers classified by ADG within both the foregut and hindgut environments [33]. Similarly, [39] did not detect alpha diversity differences in the hindgut of steers differing in feed intake and growth; however, significant differences in the relative abundances of the microbial populations were observed between efficiency groups. Although bulls sourced from different TC were fed different diets, every TC fed a total mixed ration. Similarities in the type of feed ration could explain similarities in diversity, as [40] found differences in fecal diversity of steers fed different diets.

### 4.3. Microbial Abundance

Similarly to the findings of [11], where no differences in rumen bacterial abundances regarding RFI status were found, we found only ‘X112’ to be different between RFI classification, and Succinivibrionaceae to be different between FCR classification. These findings do not reflect most results found in rumen microbiome studies where the rumen microbiome significantly differs between animals within feed efficiency classifications [41,42]. While our findings were unexpected given how essential the rumen microbiome is for feed digestion [43], these results might translate into a more favorable adoption of sample collection for microbiome analysis in cattle, given how hard it is to obtain ruminal samples.

For the fecal environment, the most abundant families across all feed efficiency classifications were Bacteroidaceae, ‘Oscillospiraceae’, ‘UBA932’, Lachnospiraceae, and Rikenellaceae (Figure S2). All five families have been found in previous research to be dominant in fibrolytic gut communities, due to their ability to breakdown recalcitrant complex carbohydrates [44,45,46,47,48]. These findings were anticipated due to the hindgut in cattle being responsible for post-ruminal degradation of cellulose and starch, highlighting its importance for feedstuff digestion [49]. There were fecal microbial taxa differences between feed efficiency classifications for all four metrics, but the families that had a significant Class effect varied for each feed efficiency classification, with RFI being the most unique. When compared to the rumen, more differences were detected in the fecal environment, regardless of the efficiency index. Previous research has also highlighted differences in microbial families in the feces of animals with diverging feed efficiency [50,51]. Interestingly, these results were obtained using different bioinformatic approaches and DNA purification methods yet agreed with the influence the hindgut has on cattle feed efficiency. For the rumen environment, results vary across microbiome feed efficiency studies in the literature, but results seem to be more agreeable across studies for the fecal environment.

These findings suggest that it may be possible to use the fecal environment for microbial biomarkers that are reflective of an animal’s level of feed efficiency, regardless of which feed efficiency metric one prefers. Fecal microbial biomarkers may allow producers to make earlier breeding selection decisions for feed efficiency in their herd. In addition, the collection of fecal samples is much more practical due to the limitation of rumen sampling. Such limitations can also decrease the number of animals available for microbiome studies in a research setting. Non-invasive esophageal tubing was utilized in the current study, but more invasive techniques such as rumenocentesis or cannulation offer more controlled rumen positioning [52], but none are feasible on a beef producer level due to the need of specialized equipment and training. Additionally, rumen contents can be collected at harvest, but prior to harvest, cattle are fasted causing a reduction in nutrients that can influence changes in their microbial population [53]. Thus, collecting feces allows for a more constant and affordable sample collection, facilitating adoption of this kind of material as microbiome selection tool. However, given that there is no consensus on which feed efficiency metric should be accepted, we do not have a consensus on which microorganisms are important in the fecal microbial population, since it differs for each metric.

## 5. Conclusions

Collecting individual feed intake data, as well as ruminal contents, is expensive and not practical for most beef producers or even in some research settings. On the other hand, collection of fecal samples is more feasible in most cases. This study identified multiple microbial families related to feed efficiency in the fecal environment of Angus bulls classified by RFI, RADG, FCR, and AFCR. Specifically, the families Lachnospiraceae, Oscillospiraceae, UBA932, Peptostreptococcaceae, Borkfalkiaceae, Enterobacteriaceae, and Eggerthellaceae were found to be significantly different in multiple feed efficiency metrics.

Using the fecal microbiome as a biomarker for feed efficiency offers a promising approach to identify the most efficient animals. However, the definition of feed efficiency must first be standardized, as the chosen metric will determine which microbial taxa are most relevant. This study advances the understanding of microbiome–efficiency relationships in cattle, but the results should be interpreted within the experimental context: efficiency status was assigned within each contemporary group. This within-group classification may have constrained broader interpretations, since animals deemed efficient on one farm may not rank similarly at the population level. Ongoing research by our group will address this limitation by evaluating microbiome–efficiency relationships across herds and production environments.

## Figures and Tables

**Table 1:** Average dry matter intake (DMI), average daily gain (ADG), and feed conversion (F:G) of Angus bulls during feed efficiency testing classified by residual feed intake (RFI), residual average daily gain (RADG), feed conversion ratio (FCR), and adjusted feed conversion ratio (AFCR).

	DMI, kg			ADG, kg/d			F:G		
Efficiency Classification ^1^	Mean	SEM ^2^	*p*-Value ^3^	Mean	SEM	*p*-Value	Mean	SEM	*p*-Value
RFI			*p* < 0.01			*p* = 0.04			*p* = 0.08
High	12.57 ^a^	0.161		1.70 ^a,b^	0.044		7.53	0.702	
Medium	10.58 ^b^	0.096		1.66 ^a^	0.026		6.75	0.263	
Low	9.20 ^c^	0.163		1.76 ^b^	0.044		5.31	0.723	
RADG			*p* = 0.66			*p* < 0.01			*p* < 0.01
High	10.50	0.214		2.04 ^a^	0.036		5.18 ^a^	0.667	
Medium	10.55	0.146		1.67 ^b^	0.025		6.40 ^a^	0.242	
Low	10.40	0.212		1.23 ^c^	0.036		10.23 ^b^	0.648	
FCR			*p* < 0.01			*p* < 0.01			*p* < 0.01
High	11.03 ^a^	0.193		1.28 ^a^	0.034		10.53 ^a^	0.647	
Medium	10.65 ^a^	0.130		1.69 ^b^	0.021		6.40 ^b^	0.666	
Low	9.43 ^b^	0.195		1.93 ^c^	0.034		4.89 ^b^	0.242	
AFCR			*p* < 0.01			*p* < 0.01			*p* < 0.01
High	10.17 ^a^	0.202		1.22 ^a^	0.033		10.22 ^a^	0.649	
Medium	10.67 ^b^	0.136		1.68 ^b^	0.022		6.42 ^b^	0.244	
Low	10.07 ^a^	0.206		2.01 ^c^	0.034		5.05 ^b^	0.669	

^1^ Efficiency groups were classified into High (upper 10%), Medium (mid 80%), and Low (lower 10%). ^2^ Standard error of the mean. ^3^ *p*-value of efficiency main effect. ^a,b,c^ Means within each feed efficiency column with different letters differ (*p* ≤ 0.05).

**Table 2:** Families found to be significantly different (*p* ≤ 0.05) within the rumen and feces of bulls classified for each feed efficiency metric.

	Feed Efficiency Metric											
	RFI ^1^			RADG ^2^			FCR ^3^			AFCR ^4^		
	High	Med.	Low	High	Med.	Low	High	Med.	Low	High	Med.	Low
**Family**	**Rumen**											
X112	0.85 ^a^	0.73 ^b^	0.68 ^b^									
Succinivibrionaceae							5.48 ^a^	3.79 ^b^	4.43 ^a,b^			
**Family**	**Feces**											
Lachnospiraceae	11.60 ^a^	10.05 ^b^	9.65 ^b^				10.94 ^x^	9.96 ^y^	9.49 ^y^			
Acutalibacteraceae	4.66 ^a^	4.98 ^a^	5.48 ^b^									
Treponemataceae	1.77 ^a^	2.70 ^b^	2.77 ^a,b^									
Fam. Class Clostridia_258483	0.87 ^a^	0.78 ^a,b^	0.65 ^b^									
CAG-382	0.39 ^a^	0.35 ^a,b^	0.29 ^b^									
Coprobacillaceae	0.38 ^a^	0.27 ^b^	0.27 ^a,b^									
UBA1242	0.23 ^a^	0.29 ^b^	0.32 ^b^									
Anaeroplasmataceae	0.23 ^a^	0.10 ^b^	0.09 ^b^									
UBA644	0.11 ^a^	0.15 ^b^	0.16 ^b^									
Fam. from Order RFN20	0.15 ^a^	0.12 ^b^	0.11 ^b^									
CAG-826	0.09 ^a,b^	0.07 ^a^	0.14 ^b^									
Oscillospiraceae				12.92 ^a^	12.56 ^a,b^	11.96 ^b^	11.95 ^a^	12.53 ^a,b^	13.15 ^b^	11.95 ^a^	12.55 ^a,b^	12.90 ^b^
UBA932				10.44 ^a^	9.96 ^a^	8.88 ^b^	8.67 ^a^	9.95 ^b^	10.59 ^b^	8.99 ^a^	9.98 ^b^	10.43 ^b^
Peptostreptococcaceae				3.16 ^a^	3.07 ^a^	3.99 ^b^	4.03 ^a^	3.07 ^b^	3.12 ^b^	3.96 ^a^	3.07 ^b^	3.12 ^b^
Borkfalkiaceae				0.80 ^a^	0.68 ^a,b^	0.62 ^b^	0.62 ^a^	0.68 ^a,b^	0.80 ^b^			
Methanobacteriaceae							0.89 ^a^	0.70 ^a,b^	0.53 ^b^			
Enterobacteriaceae_A				0.14 ^a^	0.15 ^a^	0.58 ^b^	0.41 ^a^	0.17 ^b^	0.14 ^b^			
Eggerthellaceae				0.10 ^a^	0.11 ^a,b^	0.13 ^b^	0.13 ^a^	0.11 ^a,b^	0.09 ^b^			
Turicibacteraceae										1.09 ^a^	0.84 ^b^	0.88 ^a,b^

^1^ Residual feed intake. ^2^ Residual average daily gain. ^3^ Feed conversion ratio. ^4^ Adjusted feed conversion ratio. Each metric was assigned one color to improve visualization. ^a,b^ Means with different letters differed (*p* ≤ 0.05). ^x,y^ Means with different letters tended to differ (*p* ≤ 0.10).

## Data Availability

The original contributions presented in this study are included in the article/Supplementary Material. Further inquiries can be directed to the corresponding author.

## Supplementary Materials

The following supporting information can be downloaded at https://www.mdpi.com/article/10.3390/ani15233416/s1, Table S1. Total mixed ration chemical analysis fed to bulls at each testing center on a dry matter (DM) basis. Table S2. Alpha diversity indexes in the ruminal environment of bulls classified by each feed efficiency metric. Table S3. Alpha diversity indexes in the fecal environment of bulls classified by each feed efficiency metric. Figure S1. Regression of each feed efficiency metric versus daily feed cost and daily dollar gain (values in US Dollars and British Imperial System). Figure S2. Relative microbial abundance of the top 10 most abundant families in the fecal environment of bulls classified by each feed efficiency metric.

## Abbreviations

The following abbreviations are used in this manuscript:

RFI	Residual feed intake
RADG	Residual average daily gain
FCR	Feed conversion ratio
AFCR	Adjusted feed conversion ratio
GIT	Gastrointestinal tract
DNA	Deoxyribonucleic acid
PCR	Polymerase chain reaction
DMI	Dry matter intake
DM	Dry matter
ADG	Average daily gain
