# Supplementary material for: Microbial Biomarkers Differ for Various Feed Efficiency Metrics in Beef Cattle
Source: Animals (Basel). 2025 Nov 26;15(23):3416. doi: 10.3390/ani15233416 (PMC12691302; doi:10.3390/ani15233416)

Supplemental Table S1. Total mixed ration chemical analysis fed to bulls at each testing center on a dry matter (DM) basis.

	Testing Center Contemporary Group									
	A	B	C	D	E	F	G	H	I	J
Chemical analysis[1], % DM										
DM	89.8	54.0	62.8	58.5	53.0	59.2	44.6	41.7	56.9	39.6
Crude protein	12.9	13.8	13.8	13.9	13.8	16.0	12.9	13.8	12.4	13.2
ADF[2]	26.2	28.8	26.0	31.6	30.3	17.3	20.2	18.2	26.4	31.9
NDF[3]	36.2	44.7	33.8	46.1	49.6	29.1	31.0	28.7	40.9	50.6
Lignin	6.15	4.04	5.18	6.52	5.30	2.82	2.86	2.41	5.32	3.80
Crude fat	6.51	3.27	4.03	3.03	3.31	5.29	4.29	4.56	2.99	3.25
Ash	9.85	7.34	7.17	8.99	9.74	5.17	7.62	6.86	6.71	7.33
Calcium	0.54	0.52	0.82	1.12	0.59	0.85	0.61	0.76	1.02	0.67
Phosphorous	0.61	0.58	0.28	0.26	0.50	0.48	0.54	0.50	0.33	0.34
Magnesium	0.41	0.29	0.21	0.21	0.23	0.26	0.24	0.22	0.24	0.18
Potassium	1.34	1.62	1.48	1.71	1.75	0.99	1.06	1.14	1.56	1.67
Sodium	0.32	0.15	0.05	0.06	0.22	0.19	0.16	0.14	0.12	0.06
Trace minerals, PPM										
Iron	1,022	391	299	458	845	173	486	271	235	344
Manganese	121.0	50.0	32.0	42.0	63.0	60.0	63.0	50.5	80.5	90.0
Zinc	79.0	69.0	22.0	23.0	99.0	94.0	68.0	108.0	99.0	75.0
Copper	25.0	14.0	7.0	7.0	18.0	55.0	13.0	13.5	31.0	22.0
TDN[4]	66.8	65.4	68.5	59.1	59.8	77.2	72.0	75.4	65.6	64.8
Net energy maintenance, MJ/kg	6.82	6.55	7.10	5.53	5.63	8.49	7.65	8.21	6.64	6.46
Net energy gain, MJ/kg	4.24	4.06	4.52	3.14	3.23	5.72	4.98	5.53	4.06	3.96
Non-fiber carbohydrates, % DM	36.6	33.8	43.9	33.1	25.6	46.1	44.9	48.0	38.6	27.4

[1]Cumberland Valley Analytical Services, Waynesboro, PA.

[2]ADF; Acid Detergent Fiber.

[3]NDF; Neutral Detergent Fiber.

[4]TDN; Total Digestible Nutrients.

Supplemental Table S2. Alpha diversity indexes in the ruminal environment of bulls classified by each feed efficiency metric.

	Rumen Alpha Diversity								
	Richness[1]			Evenness[2]			Diversity[3]		
Efficiency Classification[4]	Mean	SEM[5]	*P*-value	Mean	SEM	*P*-value	Mean	SEM	*P*-value
RFI			*P* = 0.23			*P* = 0.48			*P* = 0.63
High	867.72	41.169		0.87	0.004		8.39	0.093	
Medium	863.42	37.706		0.87	0.002		8.44	0.073	
Low	832.42	41.141		0.87	0.005		8.40	0.094	
RADG			*P* = 0.08			*P* = 0.30			*P* = 0.26
High	830.72[x]	40.618		0.86	0.005		8.34	0.095	
Medium	860.97[x,y]	37.058		0.87	0.002		8.45	0.075	
Low	886.47[y]	40.419		0.87	0.005		8.46	0.094	
FCR			*P* = 0.02			*P* = 0.11			*P* = 0.04
High	849.54[a,b]	40.998		0.86	0.005		8.35	0.095	
Medium	867.14[a]	37.615		0.87	0.002		8.46	0.077	
Low	816.96[b]	41.053		0.87	0.005		8.33	0.096	
AFCR			*P* = 0.30			*P* = 0.47			*P* = 0.38
High	857.48	41.044		0.87	0.005		8.42	0.094	
Medium	864.03	37.569		0.87	0.002		8.45	0.075	
Low	835.08	41.040		0.87	0.005		8.36	0.095	

[1]Number of amplicon sequence variances.

[2]Pielou's Evenness.

[3]Shannon Diversity Index.

[4]Efficiency groups are classified into high (upper 10%), Medium (mid 80%), and Low (lower 10%).

[5]Standard error of the mean.

[a,b]Means within each feed efficiency column with different letters differ ($P \leq 0.05$).

[x,y]Means within each feed efficiency column with different letters tend to differ ($P \leq 0.10$).

Supplemental Table S3. Alpha diversity indexes in the fecal environment of bulls classified by each feed efficiency metric.

	Fecal Alpha Diversity								
	Richness[1]			Evenness[2]			Diversity[3]		
Efficiency Classification[4]	Mean	SEM[5]	*P*-value	Mean	SEM	*P*-value	Mean	SEM	*P*-value
RFI			*P* = 0.12			*P* = 0.37			*P* = 0.11
High	686.71[x]	35.599		0.88	0.003		8.19[x]	0.071	
Medium	702.41[x,y]	33.605		0.88	0.002		8.24[x,y]	0.058	
Low	723.09[y]	33.534		0.88	0.003		8.32[y]	0.071	
RADG			*P* = 1.0			*P* = 0.45			*P* = 0.56
High	706.62	35.959		0.88	0.003		8.26	0.072	
Medium	705.71	34.119		0.88	0.002		8.25	0.059	
Low	705.07	35.948		0.88	0.003		8.20	0.072	
FCR			*P* = 0.14			*P* = 0.04			*P* = 0.02
High	683.21	35.923		0.87[a]	0.003		8.13[a]	0.071	
Medium	707.35	34.128		0.88[b]	0.003		8.26[b]	0.059	
Low	714.81	35.958		0.88[a,b]	0.002		8.28[b]	0.072	
AFCR			*P* = 0.77			*P = 0.63*			*P* = 0.56
High	700.44	35.970		0.88	0.003		8.21	0.071	
Medium	707.66	34.218		0.88	0.002		8.25	0.059	
Low	713.32	36.113		0.88	0.003		8.27	0.072	

[1]Number of amplicon sequence variances.

[2]Pielou's Evenness.

[3]Shannon Diversity Index.

[4]Efficiency groups are classified into high (upper 10%), Medium (mid 80%), and Low (lower 10%).

[5]Standard error of the mean.

[a,b]Means within each feed efficiency column with different letters differ ($P \leq 0.05$).

[x,y]Means within each feed efficiency column with different letters tend to differ ($P \leq 0.10$).

Supplemental Figure S1. Regression of each feed efficiency metric versus daily feed cost and daily dollar gain (values in US Dollars and British Imperial System).

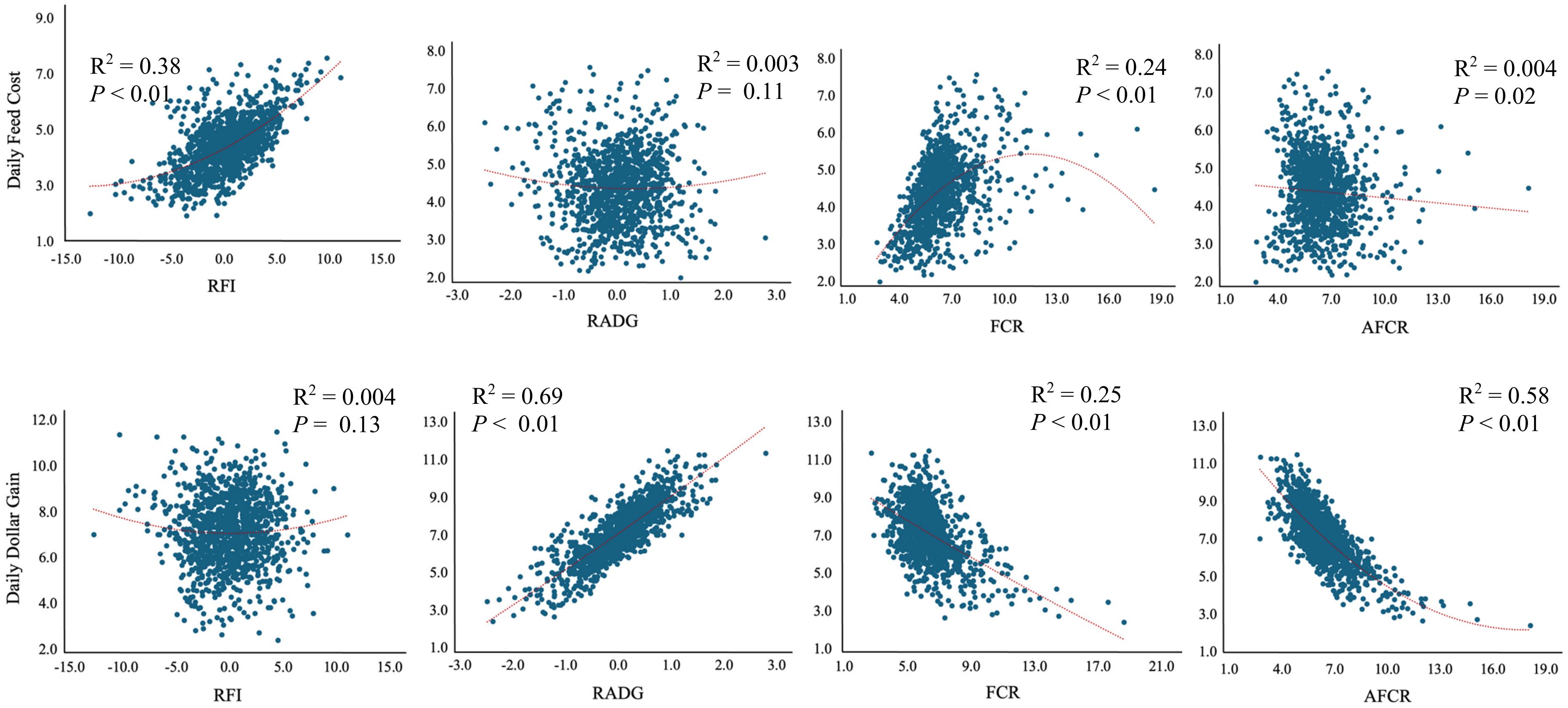

Supplemental Figure S2. Relative microbial abundance of the top 10 most abundant families in the fecal environment of bulls classified by each feed efficiency metric.

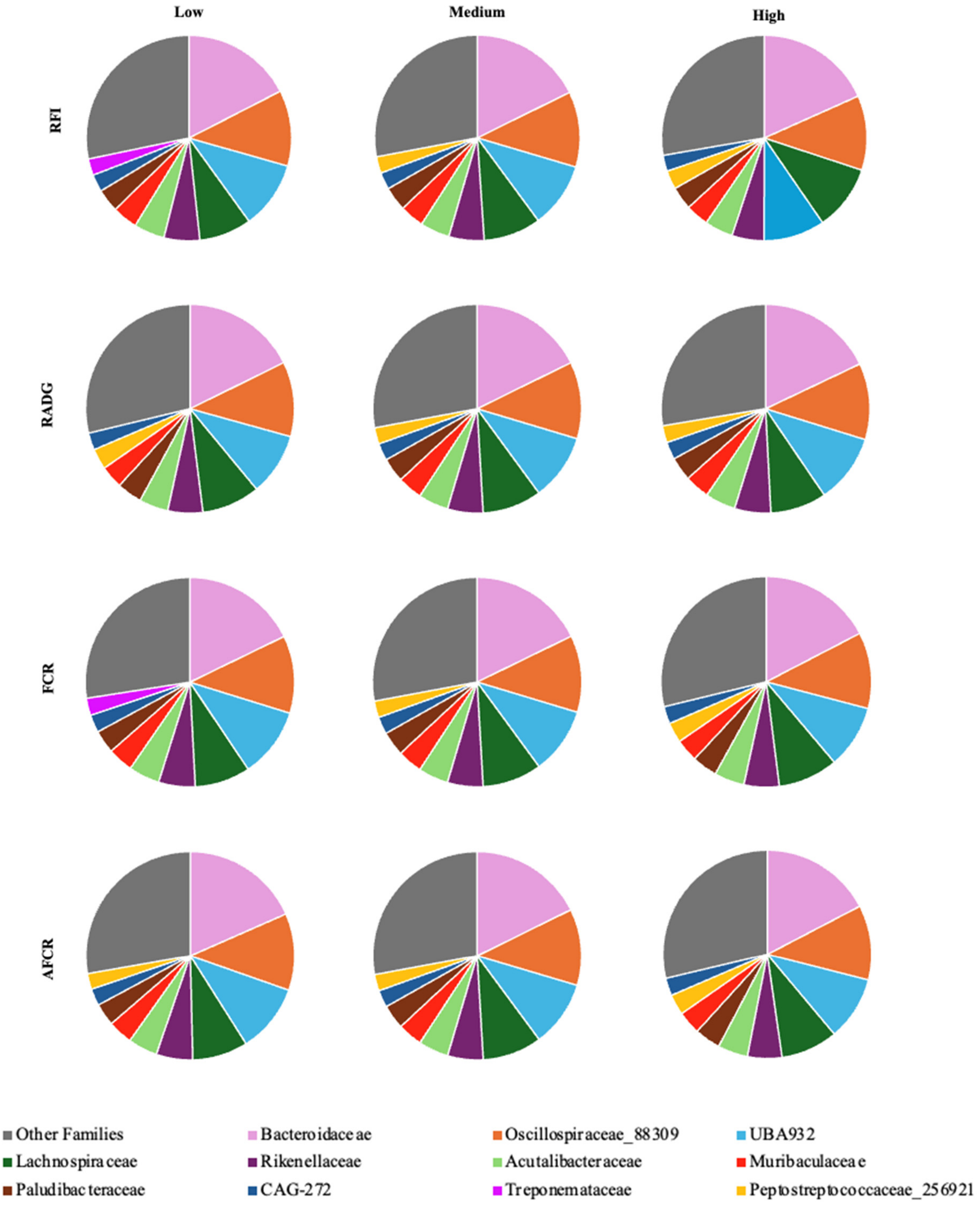

Supplement: Supplementary file 1 [file animals-15-03416-s001.zip › animals-3924242-supplementary.pdf]
